# Performance of the LDBio *Aspergillus* ICT lateral flow assay and western blot for diagnosing chronic pulmonary aspergillosis in post-tuberculosis patients: a prospective study from South India

**DOI:** 10.1128/spectrum.03847-25

**Published:** 2026-03-06

**Authors:** Arghadip Samaddar, Payel Pramanik, Harsha Voleti, J. S. Akshata, S. Nagarathna, K. Thennarasu, C. Nagraja

**Affiliations:** 1 Department of Neuromicrobiology, National Institute of Mental Health and Neuro Sciences, Bengaluru, Karnataka, India; 2 Department of Pulmonary Medicine, SDS Tuberculosis Research Centre and Rajiv Gandhi Institute of Chest Diseases 485793 , Bengaluru, Karnataka, India; 3 Department of Biostatistics, National Institute of Mental Health and Neuro Sciences, Bengaluru, Karnataka, India; Mayo Foundation for Medical Education and Research, Rochester, Minnesota, USA

**Keywords:** chronic pulmonary aspergillosis, immunochromatographic test, lateral flow assay, western blot, post-tuberculosis lung disease

## Abstract

Chronic pulmonary aspergillosis (CPA) is an important sequela of pulmonary tuberculosis (PTB), particularly in high-burden countries such as India. This study evaluated the diagnostic performance of the LDBio *Aspergillus* ICT lateral flow assay and western blot (WB) for CPA among post-TB patients in South India. In this prospective study (July 2023 to June 2025) conducted in Bengaluru, India, we enrolled adults with a history of PTB and persistent respiratory symptoms. CPA was diagnosed using the modified European Society for Clinical Microbiology and Infectious Diseases (ESCMID)-European Confederation of Medical Mycology (ECMM)-European Respiratory Society (ERS) criteria. All participants underwent comprehensive clinical evaluation; direct microscopy and culture of respiratory secretions; and testing with serum *Aspergillus*-specific IgG ELISA, ICT, WB, and galactomannan (GM). Of 132 post-TB patients, 107 (81%) were classified as CPA and 25 as non-CPA; 70 control sera were also included. ICT and WB were positive in 74% and 84% of CPA cases, respectively, outperforming ELISA (68%) and GM (30%). WB showed the highest sensitivity (84%, 95% CI: 75.8–90.5), whereas ICT demonstrated the highest specificity (94.7%, 95% CI: 88.1–98.3). Combining ICT and WB improved diagnostic accuracy (87.1%, 95% CI: 81.7–91.4) and yielded the highest discriminative power (Youden’s index 0.805). WB displayed robust band-based differentiation, with strong reactivity in seropositive CPA. The agreement between ICT and WB was substantial (κ = 0.82). Latent class analysis estimated sensitivities of 92% (95% CI: 84–97) for ICT and 87% (95% CI: 77–94) for WB. These findings indicate that LDBio ICT and WB are robust diagnostic tools for CPA in post-TB patients and that their combined use offers optimal accuracy in resource-limited, high-burden settings.

**IMPORTANCE:**

Chronic pulmonary aspergillosis (CPA) is a serious but often overlooked complication in people who have previously been treated for tuberculosis. Many of these patients continue to struggle with cough, breathlessness, and lung damage; however, routine tests often fail to explain their symptoms. This study shows that CPA is extremely common in this high-risk group and demonstrates that two simple blood tests, the LDBio Aspergillus immunochromatographic test and western blot, can reliably identify the disease. Western blot detects CPA even in patients who typically test negative on standard methods, and using both tests together markedly improves accuracy. Because these tools are quick, inexpensive, and easy to perform, they offer a practical way to diagnose CPA earlier, especially in settings where advanced testing is unavailable. Early diagnosis allows timely antifungal treatment, which can greatly reduce illness and prevent unnecessary suffering among the growing number of TB survivors.

## INTRODUCTION

Chronic pulmonary aspergillosis (CPA) is a progressive, destructive lung disease caused predominantly by *Aspergillus* species, most commonly *A. fumigatus* (1). It typically develops in individuals with pre-existing structural lung abnormalities, particularly following pulmonary tuberculosis (PTB), chronic obstructive pulmonary disease (COPD), sarcoidosis, or other cavitary lung disorders (2, 3). Among these, healed or residual PTB cavities represent the most frequent predisposing condition for CPA, especially in countries with a high TB burden, such as India (4, 5). The coexistence of post-TB lung disease and CPA poses a significant clinical challenge, as their overlapping symptoms (chronic cough, hemoptysis, weight loss, and fatigue) often result in delayed or missed diagnosis (6).

Globally, the prevalence of CPA is estimated to exceed three million cases, with approximately 1.2 million occurring as sequelae of PTB (7). India contributes substantially to this global burden, with an estimated 300,000 new cases annually (8), largely due to its high TB incidence and large population of susceptible individuals. Despite this, CPA remains underdiagnosed and underreported, primarily because of limited clinical awareness, overlapping radiological findings, and restricted access to reliable diagnostic tools in resource-limited settings (9).

Diagnosis of CPA requires a combination of chronic respiratory symptoms, characteristic radiological findings (such as cavities, fungal balls, nodules, or pleural thickening), and microbiological or immunological evidence of *Aspergillus* infection (10). Among these, detection of *Aspergillus*-specific antibodies, particularly IgG, is central to diagnosis, as fungal culture and histopathology are often insensitive or unavailable (10). The classical method for antibody detection, the precipitin assay (Ouchterlony double diffusion or counterimmunoelectrophoresis), is widely used but limited by long turnaround times, poor inter-laboratory reproducibility, and lack of standardization (11, 12). Alternative serological assays such as indirect hemagglutination and enzyme-linked immunosorbent assays (ELISA)/enzyme immunoassay (EIA) are commercially available (13), but their performance varies across platforms, necessitating population-specific recalibration of cutoff values to achieve optimal diagnostic accuracy (14). These limitations restrict their routine use in low- and middle-income countries, where the burden of TB and CPA is greatest and where affordable, rapid diagnostics are urgently needed.

Recently, the LDBio *Aspergillus* immunochromatographic test (ICT) IgG/IgM lateral flow assay (LFA) has emerged as a rapid, point-of-care alternative for CPA diagnosis. This test detects anti-*Aspergillus* antibodies in serum within 30 min and offers advantages of simplicity, speed, and minimal technical expertise (15). It meets the World Health Organization’s ASSURED criteria (affordable, sensitive, specific, user-friendly, rapid and robust, equipment-free, and deliverable to end users), making it well suited for use in resource-limited settings (15). Although several studies have demonstrated promising diagnostic performance, data from high TB-burden regions, such as India, remain limited.

The *Aspergillus* western blot (WB) IgG assay, which employs immunoblotting to detect at least two of four key *Aspergillus*-specific antibodies, has also shown good diagnostic performance across various forms of CPA (12). In contrast to ICT, which provides qualitative results, WB is a semi-quantitative antibody detection method that may facilitate estimation of *Aspergillus*-specific IgG levels based on band number and reactivity intensity. This is particularly useful in settings where automated ELISA platforms are unavailable. High baseline *Aspergillus* IgG titers have been associated with better clinical response (16), while declining titers indicate therapeutic improvement, and rising levels occurring after therapy discontinuation suggest relapse (17). Few studies have evaluated WB performance in populations at high risk for CPA, such as post-TB patients. Given variations in underlying disease prevalence, immune status, and environmental exposure, regional validation of these assays is essential before routine clinical adoption.

The present study, therefore, aimed to determine the prevalence of CPA among HIV-negative, *Mycobacterium tuberculosis*-negative post-TB patients with persistent respiratory symptoms after completion of anti-tubercular therapy (ATT) and to evaluate the diagnostic performance of the LDBio *Aspergillus* ICT and WB assays for CPA detection in this high-risk cohort.

## MATERIALS AND METHODS

### Study design

We conducted a prospective study between July 2023 and June 2025 in collaboration with the SDS Tuberculosis Research Centre and Rajiv Gandhi Institute of Chest Diseases (RGICD), a government-run autonomous tertiary care super specialty referral institution in Bengaluru, Karnataka, India.

### Ethical considerations

The study protocol was approved by the Institute Ethics Committee of both National Institute of Mental Health and Neuro Sciences (NIMHANS) and RGICD (Ref. NIMHANS/IEC [BS & NS DIV.]/43^rd^ MEETING/2023 and SDS/EC/01/2023-24). Written informed consent was obtained from all the participants. The results are reported following the Standards for Reporting of Diagnostic Accuracy Studies (STARD) guidelines (18). The study complies with the Declaration of Helsinki involving human subjects.

### Study participants

We enrolled consecutive patients aged ≥18 years with a documented history of microbiologically confirmed PTB who presented with persistent respiratory symptoms following the completion of ATT. Control sera were obtained from individuals whose samples had been submitted to the Neuromicrobiology laboratory at NIMHANS for routine diagnostic evaluation and were matched to the CPA patient cohort by age range and gender ratio. Individuals with evidence of fungal infection or with any condition known to predispose to CPA were excluded.

### Eligibility criteria

CPA was diagnosed according to the modified European Society for Clinical Microbiology and Infectious Diseases (ESCMID)-European Confederation of Medical Mycology (ECMM)-European Respiratory Society (ERS) guidelines (10). A diagnosis of CPA required fulfillment of all the following criteria: (i) presence of respiratory or systemic symptoms lasting ≥3 months; (ii) chest computed tomography (CT) findings showing one or more of the following abnormalities: one or more cavities with surrounding fibrosis, fungal ball, infiltrates, consolidation, pleural thickening adjacent to a cavity, nodules, or volume loss; (iii) evidence of *Aspergillus* infection, defined as a positive *Aspergillus* IgG antibody test and/or detection of *Aspergillus* hyphae or isolation of *Aspergillus* species from respiratory secretions, or serum or bronchoalveolar lavage fluid (BALF) galactomannan index (GMI) ≥1.65 and ≥ 2.5, respectively (19); and (iv) exclusion of alternative diagnoses (active PTB or non-tuberculous mycobacterial infection, histoplasmosis, sarcoidosis, idiopathic interstitial pneumonia, vasculitis, or lung cancer) (9, 19, 20). Chest imaging findings were independently reviewed by two radiologists and two pulmonologists. Discrepancies were resolved through discussion, and final interpretations were reached by consensus. Participants who did not meet the above criteria were categorized as non-CPA post-TB lung disease.

We excluded individuals who (i) did not provide informed consent; (ii) had received antifungal therapy for > 3 weeks within the past 3 months; (iii) were on immunosuppressive therapy, had human immunodeficiency virus (HIV) infection, or any other immunodeficiency; and (iv) were pregnant.

### CPA classification

CPA was categorized into four subtypes based on the guidelines of the ESCMID, ECMM, and ERS (10). A simple aspergilloma (SA) was defined as a single pulmonary cavity containing a fungal ball, with serological or microbiological evidence of *Aspergillus* spp., occurring in an immunocompetent patient who has minor or no symptoms and demonstrates no radiological progression over at least 3 months of observation. Chronic cavitary pulmonary aspergillosis (CCPA) was characterized by one or more thin- or thick-walled pulmonary cavities that contained aspergillomas or irregular intraluminal material, accompanied by serological or microbiological evidence of *Aspergillus* spp., significant pulmonary and/or systemic symptoms, and clear radiological progression (new cavities, increasing pericavitary infiltrates, or advancing fibrosis) over a minimum of 3 months. Chronic fibrosing pulmonary aspergillosis (CFPA) was defined as severe fibrotic destruction, typically presenting as consolidation, affecting at least two lung lobes as a complication of CCPA, causing substantial loss of lung function. Subacute invasive aspergillosis (SAIA) is an invasive aspergillosis developing over 1–3 months, primarily in mildly immunocompromised patients, characterized by variable radiological features (cavitation, nodules, and progressive consolidation with abscess formation) and supported by microbiological evidence, including positive *Aspergillus* galactomannan (GM) antigen in blood or respiratory fluids. No cases of isolated *Aspergillus* nodules were identified in the present study.

### Study procedures

All study participants underwent detailed clinical assessment, high-resolution computed tomography (HRCT) of the chest, and laboratory investigations, including hematological tests, sputum smear microscopy, cartridge-based nucleic acid amplification test (GeneXpert MTB/RIF; Cepheid, Sunnyvale, CA, USA), Mycobacterial Growth Indicator Tube (MGIT) culture, direct microscopy and fungal culture of sputum or BALF samples, measurement of serum *Aspergillus*-specific IgG, and serum GM antigen detection.

### Serum *Aspergillus*-specific IgG ELISA

We measured serum *Aspergillus*-specific IgG levels using the Platelia *Aspergillus* IgG ELISA (Bio-Rad, Marnes-la-Coquette, France) (21). Serum samples were diluted 1:400 and added to microplate wells coated with purified recombinant *Aspergillus* antigen. A peroxidase-labeled secondary antibody was then added to each well. Optical density (OD) was measured at 450/620 nm using a spectrophotometer. IgG titers (arbitrary units per milliliter, AU/mL) were calculated based on calibration standards ranging from 0 to 80 AU/mL. According to the manufacturer’s guidelines, results <5 AU/mL were considered negative, 5–10 AU/mL intermediate, and ≥10 AU/mL positive. For samples exceeding 80 AU/mL, repeat testing was performed with an additional 1:5 dilution if OD < 3.0 or 1:60 dilution if OD ≥ 3.0. Patients with intermediate IgG results were classified based on corresponding sputum or BALF culture findings.

### *Aspergillus* ICT IgG/IgM LFA

Each sample was tested using the *Aspergillus* ICT IgG/IgM LFA (LDBio Diagnostics, Lyon, France) in accordance with the manufacturer’s instructions. Test kits were transported at ambient temperature and stored at 4°C. Prior to use, each batch of LFA kits (10 tests per pack) was equilibrated to room temperature. Briefly, 15 μL of serum was applied to the ICT sample pad, followed by four drops of the provided elution buffer. The results were interpreted after 20–30 min. The test was deemed positive when both the blue control (“C”) line and the black test (“T”) line were visible; it was considered negative if only the control line appeared. An equivocal result was defined by the presence of a faint or diffuse gray line below the “T” line. The equivocal results were excluded from analysis.

### *Aspergillus* WB IgG assay

Each serum sample was tested using the *Aspergillus* WB IgG kit (LDBio Diagnostics, Lyon, France) following the manufacturer’s instructions. Briefly, 1.2 mL of sample buffer was added to each channel of an incubation tray, and test strips were placed into the tray. Then, 10 μL of serum was dispensed according to the distribution plan, and the strips were incubated for 90 min under agitation. A manufacturer-supplied positive control was included in each run. After three washes with wash buffer (diluted 1:10), 1.2 mL of IgG conjugate was added to each channel, followed by a 60-min incubation with agitation. After another wash step, 1.2 mL of substrate solution was added, and the strips were incubated for approximately 60 min. The strips were washed twice with water and air-dried at room temperature for at least 15 min before interpretation. The results were compared with the positive control and classified as positive or negative according to the manufacturer’s guidelines. Four protein bands, at 16, 18–20, 22, and 30 kDa, are considered specific markers of *Aspergillus* sensitization. The test was interpreted as positive when at least two of these bands were present. The overall WB intensity was determined by summing the intensity scores (ranging from 1 to 4) of each specific band, with the final intensity categorized as weak (<2), moderate (2–4), high (5–10), or very high (>10), as described previously (12). Each test was performed in duplicate and independently read by two experts. To assess both inter- and intra-lot consistency, a panel of eight quality-control serum samples was tested.

### Serum galactomannan assay

Serum GM antigen was detected using a one-step immune-enzymatic sandwich assay on microplates (Platelia *Aspergillus* EIA, Bio-Rad Laboratories). Each assay run included a positive control, a negative control, and duplicate wells of the cutoff control. Absorbance was measured at 450 nm with a 620 nm reference filter. The GM optical density index (ODI) was calculated by comparing the optical density of each test sample to that of the cutoff control.

### Sputum and BALF investigations

Sputum samples were primarily obtained by expectoration, with a smaller number collected through sputum induction. Both sputum and BALF samples were initially liquefied using N-acetyl-L-cysteine in the presence of sodium citrate, then neutralized with phosphate-buffered saline. The processed specimens were centrifuged, and the resulting pellets were examined microscopically using calcofluor white staining with potassium hydroxide. High-volume culture (HVC) was performed for all sputum samples, with up to 1 mL of undiluted specimen plated on two Sabouraud agar plates and incubated at 30°C for up to 14 days (22). Colonies exhibiting mycelial growth were sub-cultured onto malt extract agar and Czapek-Dox agar, and *Aspergillus* species were identified based on their morphological characteristics and using matrix-assisted laser desorption/ionization-time of flight mass spectrometry (MALDI-TOF MS).

### Statistical analysis

We used the modified ESCMID/ECMM/ERS criteria for CPA (10) as the reference standard to assess the diagnostic performance of the LDBio ICT, WB, combined ICT plus WB, and GM. In addition, we conducted Latent Class Analysis (LCA) to compare the performance of the LDBio ICT and WB, considering CPA as the latent variable and not using a reference standard. Categorical variables were reported as frequencies and percentages, and continuous variables as mean with standard deviation (SD) or median with interquartile range (IQR), as appropriate. Associations between categorical variables were assessed using Fisher’s exact or chi-squared tests, while continuous variables were compared using Student’s *t*-test or Wilcoxon rank-sum test. Pearson’s chi-square test was used to compare sensitivities between independent groups. McNemar’s test was applied with Yates correction (1.0) for pairwise comparisons of sensitivity between the LDBio *Aspergillus* ICT, WB, and ELISA. Youden’s J statistic (sensitivity + specificity – 1) and diagnostic odds ratio (DOR) were calculated (23). Binomial confidence intervals (*CIs*) (95%) were calculated for sensitivity, specificity, positive and negative predictive values (PPV and NPV), positive and negative likelihood ratios (PLR and NLR), and the diagnostic odds ratio (DOR). Concordance between ICT, WB, and ELISA results was assessed using Cohen’s kappa coefficient, interpreted as slight (0–0.20), fair (0.21–0.40), moderate (0.41–0.60), substantial (0.61–0.80), or almost perfect (0.81–1.00) agreement (24). A two-tailed *P*-value <0.05 was considered statistically significant. Statistical analyses were performed using SAS version 9.3 (SAS Institute, Inc., Cary, NC) and GraphPad Prism version 10.2.3 for Windows (GraphPad Software, Boston, MA, USA).

The LCA was performed using the TAGS (Tests in the Absence of a Gold Standard) software implemented in R version 2.2 (The R Development Core Team) (25). LCA was used to characterize the relationship between the observed diagnostic variables (LDBio ICT and WB) and an unobserved latent variable (here representing CPA), under the assumption of conditional independence between the diagnostic tests. Both index tests were applied to each participant, and the results were coded as binary values (0 or 1). The 2^n^ possible result permutations were recorded in TAGS. The package estimated test sensitivities and specificities using maximum likelihood methods (Newton–Raphson and expectation–maximization), with 95% CI (corresponding to 2.5%–97.5% bootstrap *CIs* obtained from 5000 bootstrap samples). Model fit and conditional independence were evaluated using the goodness-of-fit test. A *P*-value > 0.05 and residuals randomly distributed around zero indicated adequate model fit (25).

## RESULTS

### Patient demographics

A total of 132 smear-negative, GeneXpert-negative post-TB patients with persistent respiratory symptoms were enrolled during the study period, of which 107 patients met the criteria for CPA, while 25 were diagnosed with non-CPA post-TB lung disease. None of the patients had a positive mycobacterial culture. In addition, 70 control sera were also included (Table 1). The median age of CPA patients was 50 years (IQR: 22–82), comparable to that of non-CPA (48 years) and controls (53.5 years). Males predominated among CPA cases (80/107, 75%), although the difference was not statistically significant (*P* = 0.13). Smoking (55/107, 51%) and alcohol consumption (48/107, 45%) were significantly more frequent among CPA patients than in controls (*P* = 0.015 and < 0.001, respectively) (Table 1).

**TABLE 1:** Clinical characteristics of patients and controls included in the study^*a*^^,^^*b*^^,^^*c*^

Characteristics	CPA	All controls (*n* = 95)		*P* value
		Non-CPA	Controls	
No. of patients (*n*)	107	25	70	
Male	80 (75)	14 (56)	53 (76)	0.13
Female	27 (25)	11 (44)	17 (24)	
Age (y), median (IQR)	50 (22–82)	48 (22–78)	53.5 (33–73)	0.43
Smoking	55 (51)	9 (36)	21 (30)	**0.015**
Alcoholism	48 (45)	7 (28)	10 (14)	**<0.001**
Clinical features				
Fever	33 (31)	9 (36)	NA	0.62
Cough	105 (98)	25 (100)	NA	0.50
Dyspnea	82 (77)	17 (68)	NA	0.37
Hemoptysis	60 (56)	17 (68)	NA	0.28
Chest pain	51 (48)	12 (48)	NA	0.98
Duration of illness (days), median (IQR)	224 (90–5475)	240 (90–5475)	NA	0.58
ATT duration (months), median (IQR)	6 (0.2–14)	6 (5–8)	NA	0.65
Time from end of TB therapy to recruitment (months), median (IQR)	60 (6–300)	36 (8–360)	NA	0.20
Comorbidities				
Type 2 DM	18 (17)	6 (24)	NA	0.40
Hypertension	19 (18)	5 (20)	NA	0.79
COPD	15 (14)	2 (8)	NA	0.42
Others^*d*^	12 (11)	5 (20)	NA	0.32
HRCT thorax findings				
Cavity	76 (71)	15 (60)	NA	0.28
Fungal ball	54 (51)	3 (12)	NA	**0.0005**
Pericavitary fibrosis	73 (68)	13 (52)	NA	0.13
Pericavitary infiltrates	69 (64)	11 (44)	NA	0.06
Nodules	79 (74)	8 (32)	NA	**<0.0001**
Bronchiectatic changes	63 (59)	10 (40)	NA	0.09
Pleural thickening	56 (52)	18 (72)	NA	0.07
Consolidation	29 (27)	7 (28)	NA	0.93
Emphysematous changes	10 (9)	1 (4)	NA	0.40
Fibroatelectatic bands	8 (7)	2 (8)	NA	0.93
Others^*e*^	17 (16)	7 (28)	NA	0.16
Hematological parameters				
Hemoglobin, median (IQR)	12 (7.5–16.7)	12.1 (8.7–16.5)	13.2 (11.8–14.5)	0.71
TLC (in 10^9^/L), median (IQR)	9.01 (3.3–23.1)	8.65 (3.7–22.1)	6.9 (4.8–9.3)	0.21
Neutrophil (%), median (IQR)	71 (40–93)	76 (40–88)	62 (53–72)	0.13
Lymphocyte (%), median (IQR)	20 (5–47)	19 (9–46)	26 (21–33)	0.26
Monocyte (%), median (IQR)	4 (0–15)	4 (0–10)	4 (0–12)	0.80
Eosinophil (%), median (IQR)	2 (0–26)	1 (0–6)	2 (0–4)	**0.048**
ALP (IU/L), median (IQR)	84.5 (9–266)	98 (28–299)	55 (39–84)	0.07
ALT (U/L), median (IQR)	16 (0.54–531.6)	18 (6.5–907.2)	18 (11–45)	0.94
AST (U/L), median (IQR)	18.6 (0.5–246.4)	14.4 (5.1–835.4)	16 (8–38)	0.33
Total protein (g/dL), median (IQR)	6.3 (3.9–8.4)	6.4 (5–7.9)	6.6 (4.2–8.0)	0.63
Albumin (g/dL), median (IQR)	3.2 (1.6–6.1)	3.0 (2.3–4.1)	3.8 (3.0–5.2)	0.56
A:G ratio, median (IQR)	1.0 (0.4–7.5)	1.0 (0.6–7.0)	1.4 (0.8–2.2)	0.62
Sample type				
Sputum	88 (83)	17 (68)	NA	
BALF	11 (10)	5 (20)	NA	
No respiratory samples	8 (7)	3 (12)	NA	
Serum	107 (100)	25 (100)	70 (100)	
Microbiological findings				
Direct microscopy				
No. of samples tested (*n*)	99	22	NA	
Positive	27 (28)	6 (27)	NA	
Negative	71 (72)	16 (73)	NA	
Fungal culture				
No. of samples tested (*n*)	99	22	NA	
Positive	85 (86)	6 (27)	NA	**<0.0001**
Negative	14 (14)	16 (73)	NA	
*Aspergillus* growth in culture	79 (80)	0	NA	**<0.0001**
*A. fumigatus* only	9 (9)	0	NA	
*A. flavus* only	3 (3)	0	NA	
*A. niger* only	13 (13)	0	NA	
*A. fumigatus* + other *Aspergillus* spp.^*f*^	21 (21)	0	NA	
*A. flavus* + other *Aspergillus* spp.	21 (21)	0	NA	
*A. niger* + other *Aspergillus* spp.	33 (33)	0	NA	
*Aspergillus* spp. + non-*Aspergillus* molds^*g*^	28 (28)	0	NA	
Non-*Aspergillus* molds only^*h*^	6 (6)	6 (27)	NA	
*Aspergillus* serology				
No. of samples tested (*n*)	107	25	70	
ICT +	79 (74)	5 (20)	0	**<0.0001**
Western Blot +	90 (84)	10 (40)	0	**<0.0001**
ELISA^*i*^ +	73 (68)	0	0	**<0.0001**
Median IgG conc. in AU/mL (IQR)	47.6 (−5.4 to 8724.7)	−4.03 (−5.4 to 9.7)	−3.34 (−6.7 to 2.8)	**<0.0001**
Serum GM^*j*^ +	32 (30)	5 (20)	0	0.47
Mean serum GM ODI ± SD	1.3 ± 1.1	1.2 ± 1.1	0.33 ± 0.15	**<0.0001**
BALF GM^*k*^ +	4 (36)	1 (20)	NA	0.51
Antifungal therapy after CPA diagnosis				
Itraconazole	83 (78)	NA	NA	
Outcome				
Mortality	1 (0.9)	0	NA	0.63
Survival	106 (99)	25 (100)	NA	

^*a*^ A:G, albumin:globulin; ALP, alkaline phosphatase; ALT, alanine aminotransferase; AST, aspartate aminotransferase; BALF, bronchoalveolar lavage fluid; COPD, chronic obstructive pulmonary disease; CPA, chronic pulmonary aspergillosis; DM, diabetes mellitus; GM, galactomannan; HRCT, high-resolution computed tomography; ICT, immunochromatographic test; IQR, interquartile range; IU, international units; NA, not applicable; ODI, optical density index; SD, standard deviation; TB, tuberculosis; TLC, total leukocyte count.

^*b*^ The numbers in parentheses represent percentages.

^*c*^ The *P* values in bold indicate statistical significance.

^*d*^ Other comorbid conditions in CPA include malignancy (*n* = 3), asthma and hepatitis B (*n* = 2, each), and hypothyroidism, chronic kidney disease, post-TB lung disease, rheumatoid arthritis, and cor pulmonale (*n* = 1, each). In the non-CPA group, other comorbidities include malignancy, asthma, hepatitis B, hypothyroidism, and post-TB lung disease (*n* = 1, each).

^*e*^ Other HRCT findings in CPA include ground glass opacities (*n* = 5), atelectatic changes and parenchymal calcification (*n* = 3, each), and air crescent sign, mosaic attenuation, and pleuroparenchymal fibrosis (*n* = 2, each). In non-CPA patients, other HRCT findings include ground glass opacities (*n* = 3), atelectatic changes, parenchymal calcification, empyema, and abscess (*n* = 1, each).

^*f*^ Other *Aspergillus spp*. include *A. nidulans* (*n* = 2), *A. terreus* (*n* = 1), and *A. versicolor* (*n* = 1).

^*g*^ Non-*Aspergillus* molds in CPA patients include *Penicillium* spp. (*n* = 28), *Rhizopus arrhizus* (*n* = 5), and *Fusarium oxysporum* (*n* = 2).

^*h*^ Non-*Aspergillus* molds in non-CPA patients include *Penicillium* spp. (*n* = 5) and *Fusarium oxysporum* (*n* = 1).

^*i*^ *Aspergillus *IgG concentrations ≥10 AU/mL were considered to be positive by ELISA. CPA patients with consistent and repeated *Aspergillus* IgG level of <10 AU/mL were classified as “seronegative.” These cases were diagnosed as CPA based on positive *Aspergillus *growth in sputum/BALF culture.

^*j*^ Serum samples from all CPA patients, non-CPA patients, and controls were tested for galactomannan; an optical density index cutoff of ≥1.7 was used to define positivity.

^*k*^ BALF samples from 11 CPA and 5 non-CPA patients were tested for galactomannan; an optical density index cutoff of ≥ 2.6 was used to define positivity.

### Clinical features

Cough (105/107, 98%), dyspnea (82/107, 77%), and hemoptysis (60/107, 56%) were the most common clinical manifestations. The median duration of illness was 224 days (IQR: 90–5,475) in CPA, with a median ATT duration of 6 months and a median interval of 60 months (IQR: 6–300) from completion of TB treatment to study recruitment. Hypertension (19/107, 18%), type 2 diabetes mellitus (18/107, 17%), and COPD (15/107, 14%) were the most frequent comorbidities.

Characteristic HRCT findings were highly discriminative for CPA (Table 1). Fungal balls (54/107, 51%) and nodules (79/107, 74%) were significantly more common in CPA than in non-CPA patients (3/25, 12% and 8/25, 32%, respectively). Cavities (76/107, 71%), pericavitary fibrosis (73/107, 68%), infiltrates (69/107, 64%), and bronchiectatic changes (63/107, 59%) were also observed more frequently in the CPA group. Other radiologic abnormalities, including pleural thickening, consolidation, and fibroatelectatic bands, were noted at comparable frequencies across both groups.

### Laboratory investigations

Median hemoglobin and total leukocyte counts were similar across groups. Eosinophil counts were slightly higher among CPA patients (median 2%; IQR: 0–26) compared with non-CPA (1%; IQR: 0–6; *P* = 0.048). Liver enzymes, total protein, albumin, and albumin: globulin ratio did not differ significantly between groups.

Among CPA patients, direct microscopy was positive in 27/99 (28%) samples, while fungal cultures were positive in 85/99 (86%), compared with 6/25 (27%) among non-CPA patients (*P* < 0.001). *Aspergillus* spp. were isolated in 79/99 (80%) CPA cases, with mixed species growth being common. The predominant species were *Aspergillus niger* (13/99, 13%), *Aspergillus fumigatus* (9/99, 9%), and *Aspergillus flavus* (3/99, 3%), with co-isolation of multiple *Aspergillus* spp. in 34 of 85 (40%) of positive cultures. Non-*Aspergillus* molds, including *Penicillium* spp., *Rhizopus arrhizus*, and *Fusarium oxysporum*, were detected alongside *Aspergillus* spp. in 30/107 (28%) CPA cases (Table 1).

### *Aspergillus* ICT, WB, ELISA, and GM results

*Aspergillus*-specific serology showed high diagnostic yield in CPA. The LDBio ICT and WB were positive in 79/107 (74%) and 90/107 (84%) CPA patients, respectively, while IgG ELISA was positive in 73/107 (68%) (Table 1). Median *Aspergillus* IgG concentrations were significantly higher in CPA (47.6 AU/mL; IQR: –5.4 to 8,724.7) compared with non-CPA (–4.03 AU/mL) and controls (–3.34 AU/mL; *P* < 0.0001). Among CPA cases, infection due to *A. fumigatus* was associated with significantly higher median IgG levels (314.6 AU/mL; IQR: −5.2 to 8,724.7) compared with non-*fumigatus Aspergillus* species (3.07 AU/mL; IQR −5.3 to 1,382.9; *P* = 0.00007). Serum GM was positive in 32/107 (30%) CPA cases, with a higher mean ODI compared with controls (1.3 ± 1.1 vs. 0.33 ± 0.15; *P* < 0.0001). BALF GM was positive in 4/11 (36%) CPA patients.

### Diagnostic performance of *Aspergillus* ICT, WB, and GM in CPA subtypes

The diagnostic performance of LDBio ICT, LDBio WB, and GM assays is summarized in Table 2. Overall, WB demonstrated the highest single-test sensitivity (84.1%; 95% CI: 75.8–90.5), followed by ICT (73.8%; 95% CI: 64.5–81.8). When ICT and WB were used in combination, sensitivity increased markedly to 95.8% (95% CI: 91.9–99.7), with a corresponding improvement in overall diagnostic accuracy (87.1%; 95% CI: 81.7–91.4). ICT and GM exhibited higher specificities (both 94.7%; 95% CI: 88.1–98.3) than WB (89.5%; 95% CI: 81.5–94.8). Both ICT and WB outperformed the GM assay, which showed limited sensitivity (30%; 95% CI: 21.4–39.5) and low accuracy (58%; 95% CI: 50.8–64.8). In seropositive CPA, WB achieved superior sensitivity compared with ICT (98.6% vs. 93.2%). Sensitivities declined substantially in seronegative CPA (ICT: 32.4%, WB: 53%), highlighting the diagnostic challenges in this subgroup. GM sensitivity remained low across both seropositive (38.4%) and seronegative (11.8%) CPA. Youden’s index was highest for the combined ICT-WB approach (0.805), confirming the superior discriminative ability of the dual testing model.

**TABLE 2:** Performance characteristics of LDBio *Aspergillus* ICT, WB, and galactomannan assays for the diagnosis of CPA^*a*^

Parameter	ICT	WB	ICT + WB	Galactomannan
% Sensitivity (95% CI)				
All CPA (*n* = 107)	73.8 (64.5, 81.8)	84.1 (75.8, 90.5)	95.8 (91.9, 99.7)	30 (21.4, 39.5)
Seropositive CPA^*b*^ (*n* = 73)	93.2 (84.7, 97.7)	98.6 (92.6, 99.9)	99.9 (99.3, 100)	38.4 (27.2, 50.5)
Seronegative CPA^*c*^ (*n* = 34)	32.4 (17.4, 50.5)	53 (35.1, 70.2)	68.2 (59.4, 77)	11.8 (3.3, 27.5)
% Specificity (95% CI) (all controls, *n* = 95)	94.7 (88.1, 98.3)	89.5 (81.5, 94.8)	84.7 (77.8, 91.6)	94.7 (88.1, 98.3)
PPV (95% CI) (all sera, *n* = 202)	94 (87, 97.4)	90 (83.3, 94.2)	90.1 (83.4, 94.3)	76.2 (62.5, 86)
NPV (95% CI) (all sera, *n* = 202)	76.3 (70, 81.6)	83.3 (76.3, 88.6)	84.2 (77.1, 89.4)	53.1 (49.6, 56.6)
PLR (95% CI) (all sera, *n* = 202)	14 (5.9, 33.2)	8 (4.4, 14.4)	8.1 (4.5, 14.6)	2.8 (1.5, 5.5)
NLR (95% CI) (all sera, *n* = 202)	0.28 (0.2, 0.38)	0.18 (0.11, 0.28)	0.17 (0.11-0.26)	0.78 (0.68, 0.9)
DOR (95% CI) (all sera, *n* = 202)	50 (18.7, 137.8)	45 (19.5, 103.8)	48 (20.8, 112.4)	3.6 (1.7, 7.8)
Accuracy (95% CI) (all sera, *n* = 202)	83.7 (77.8, 88.5)	86.6 (81.2, 91)	87.1 (81.7, 91.4)	58 (50.8, 64.8)
Youden’s index^*d*^	0.685	0.736	0.805	0.195

^*a*^ CI, confidence interval; CPA, chronic pulmonary aspergillosis; DOR, diagnostic odds ratio; ICT, immunochromatographic test; LFA, lateral flow assay; NLR, negative likelihood ratio; NPV, negative predictive value; PLR, positive likelihood ratio; PPV, positive predictive value; WB, western blot.

^*b*^ Aspergillus IgG concentrations ≥10 AU/mL were considered to be positive by ELISA.

^*c*^ CPA patients with consistent and repeated *Aspergillus*-specific IgG levels of <10 AU/mL by ELISA were classified as “seronegative.” These cases were diagnosed as CPA based on positive *Aspergillus *growth in sputum/BALF culture.

^*d*^ Youden's index = sensitivity + specificity − 1.

Among CPA cases with *Aspergillus* growth in culture (*n* = 79), WB demonstrated significantly higher sensitivity than ICT (75.9% vs. 64.6%, *P* < 0.0001) (Table 3). For cases with *A. fumigatus* isolation (*n* = 32), sensitivities were high for both assays (93.7% for ICT and 91% for WB), with both reaching 100% (95% CI: 66.4–100) when *A. fumigatus* was the only species isolated. In infections due to non-*fumigatus Aspergillus* species (*n* = 20), sensitivities decreased to 40% for ICT and 50% for WB. In cases where *Aspergillus* and non-*Aspergillus* molds were co-isolated (*n* = 28), WB demonstrated significantly higher sensitivity than ICT (78.6% vs. 60.7%, *P* = 0.0007). Both assays were positive in all culture-negative CPA cases (*n* = 14) and in all patients for whom sputum culture was not feasible (*n* = 8).

**TABLE 3:** Performance of LDBio *Aspergillus* ICT and WB in fungal culture-positive CPA cases^*a*^

Sputum/ BALF culture result (*n* = 99)	Number	ICT + (*n*)	WB + (*n*)	% Sensitivity (95% CI)	
				ICT	WB
All *Aspergillus* growth	79	51	60	64.6 (52.9, 75)	75.9 (65, 84.9)
*A. fumigatus*	32	28	27	93.7 (79.2, 99.2)	90.6 (75, 98)
*A. fumigatus* only	9	9	9	100 (66.4, 100)	100 (66.4, 100)
*A. fumigatus* + other *Aspergillus* spp.	21	19	18	90.5 (69.6, 98.8)	85.7 (63.7, 96.9)
Other *Aspergillus* spp.	20	8	10	40 (19.1, 63.9)	50 (27.2, 72.8)
*A. niger* only	13	5	7		
*A. flavus* only	3	1	1		
*A. nidulans* only	2	0	0		
*A. terreus* only	1	1	1		
*A. versicolor* only	1	1	1		
*Aspergillus* + non-*Aspergillus* molds^*b*^	28	17	22	60.7 (40.6, 78.5)	78.6 (59.1, 91.7)
No fungal growth	14	14	14	100 (76.8, 100)	100 (76.8, 100)

^*a*^ BALF, bronchoalveolar lavage fluid; CI, confidence interval; CPA, chronic pulmonary aspergillosis; ICT, immunochromatographic test; WB, western blot.

^*b*^ Non-*Aspergillus* molds co-isolated with *Aspergillus *spp. include *Penicillium *spp. (*n* = 24), *R. arrhizus* (*n* = 4), and *F. oxysporum* (*n* = 2).

**Fig 1:**
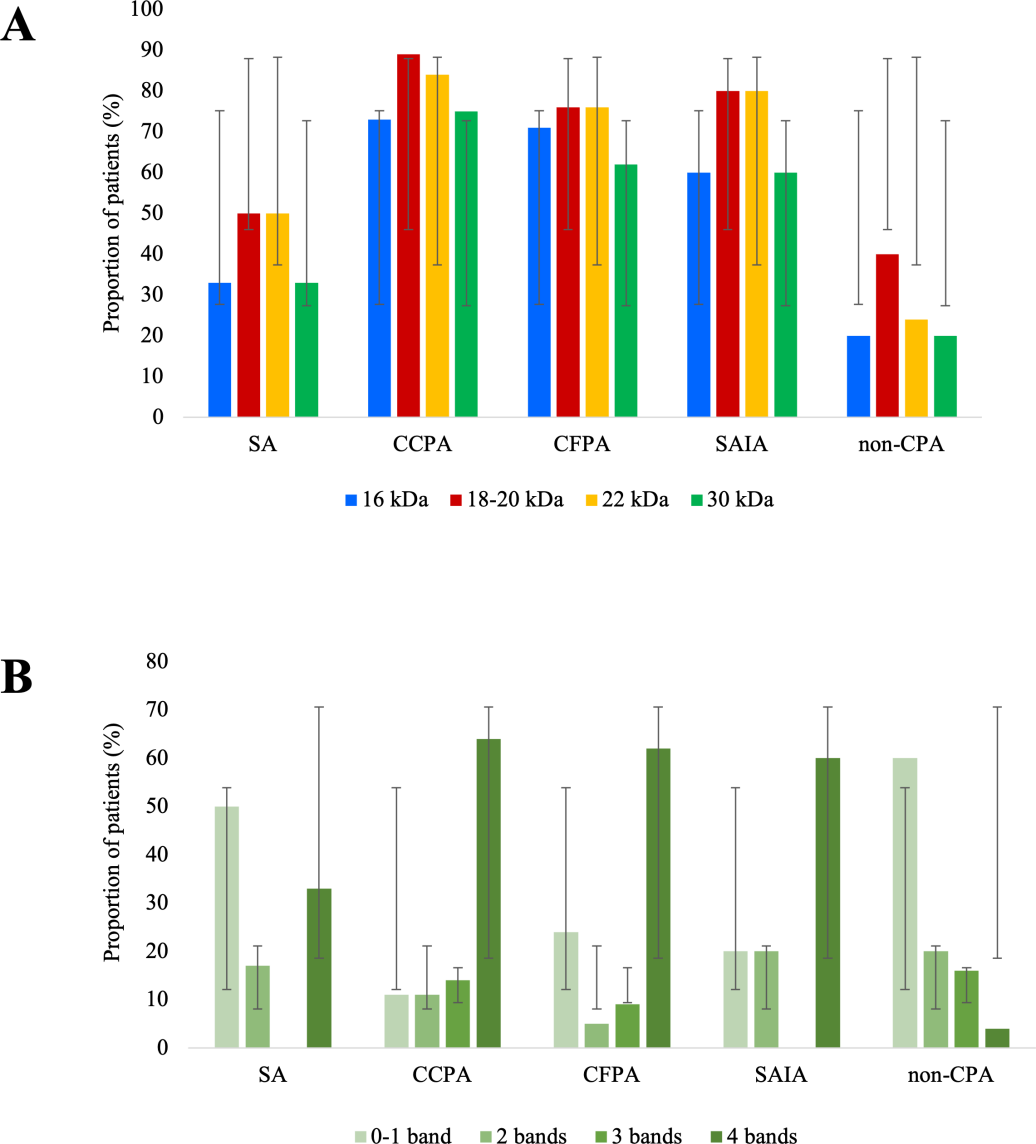
LDBio WB banding profiles and reactivity intensity across CPA subtypes and non-CPA patients. (**A**) All CPA subtypes showed a significantly higher prevalence of four distinct WB bands (16, 18–20, 22, and 30 kDa) compared with the non-CPA group (*P* < 0.0001 for all comparisons). (**B**) The proportion of patients exhibiting all four bands was substantially greater in the CPA group than in the non-CPA group (*P* < 0.0001). Abbreviations: CCPA, chronic cavitary pulmonary aspergillosis; CFPA, chronic fibrosing pulmonary aspergillosis; CPA, chronic pulmonary aspergillosis; SA, simple aspergilloma; SAIA, subacute invasive aspergillosis; WB, western blot.

Among the CPA subtypes, CCPA represented the majority (75/107, 70%), followed by CFPA (21/107, 19.7%), SA (6/107, 5.6%), and SAIA (5/107, 4.7%) (Table 4). ICT positivity was substantially higher in seropositive (68/73; 93.2%) than in seronegative CPA (11/34, 32.4%) (χ² = 51.7, *P* < 0.0001). The WB positivity was uniform across CFPA and SAIA (both 100%) and exceeded 95% among CCPA and SA. Distinct WB banding profiles were observed across CPA subtypes (Table 4, Fig. 1A). The 18–20 kDa and 22 kDa bands were most frequently recognized, present in 90/107 (84%, 95% CI: 75.7–90.3; χ² = 21.5, *P* < 0.0001) and 86/107 (80%, 95% CI: 71.6–87.2; χ² = 30.5, *P* < 0.0001) CPA patients, respectively. The 16 kDa band was detected in 75/107 (70%, χ² = 21.3, *P* < 0.0001) CPA cases. Reactivity intensity, expressed as the number of bands per sample, also differed significantly (Fig. 1B). Four-band reactivity, indicative of strong multi-antigen recognition, was more frequently observed in seropositive (64/73, 88%) than in seronegative CPA (2/34, 6%) (χ² = 65.6, *P* < 0.0001) and non-CPA patients (1/25, 4%) (χ² = 58.4, *P* < 0.0001). Conversely, weak responses (0–1 bands) were more frequent in seronegative CPA (16/34, 47%) and non-CPA patients (15/25, 60%) than in seropositive CPA (1/73, 1.4%) (χ² = 46.5, *P* < 0.0001). Mean global WB intensity (±SD) was 13.5 ± 3.05 (very high) in seropositive CPA, 4.4 ± 4.3 (high) in seronegative CPA, and 3.4 ± 4.2 (moderate) in non-CPA cases, with a highly significant difference between seropositive CPA and non-CPA (Mann–Whitney U = 101, *P* < 0.0001). Median (IQR) global intensities followed a similar pattern (seropositive: 15 [3–16]; seronegative: 5 [0–13]; non-CPA: 2 [0–16]; Kruskal-Wallis χ² = 78.1, *P* < 0.0001). Serum GM assay was positive in 32/107 (30%) CPA cases, with the highest reactivity in SAIA (3/5, 60%), followed by CCPA (24/75, 32%) and CFPA (5/21, 24%), and was absent in SA. GM positivity did not differ significantly between CPA and non-CPA groups (χ² = 0.98, *P* = 0.321).

**TABLE 4:** Summary of LDBio *Aspergillus* ICT, WB, and GM test results, along with WB banding patterns in different CPA subtypes^*a*^

Test	Seropositive CPA (*n* = 73)				Seronegative CPA^*b*^ (*n* = 34)				Total CPA (%) (*n* = 107)	Non-CPA (%) (*n* = 25)
	SA (%) (*n* = 3)	CCPA (%) (*n* = 54)	CFPA (%) (*n* = 12)	SAIA (%) (*n* = 4)	SA (%) (*n* = 3)	CCPA (%) (*n* = 21)	CFPA (%) (*n* = 9)	SAIA (%) (*n* = 1)		
GM +	0	21 (39)	4 (33)	3 (75)	0	3 (14)	1 (11)	0	32 (30)	5 (20)
ICT +	2 (67)	52 (96)	11 (92)	3 (75)	0	8 (38)	3 (33)	0	79 (74)	5 (20)
WB +	3 (100)	53 (98)	12 (100)	4 (100)	0	14 (67)	4 (33)	0	90 (84)	10 (40)
WB banding patterns										
16 kDa	2 (67)	48 (89)	12 (100)	3 (75)	0	7 (33)	3 (33)	0	75 (70)	5 (20)
18-20 kDa	3 (100)	53 (98)	12 (100)	4 (100)	0	14 (67)	4 (44)	0	90 (84)	10 (40)
22 kDa	3 (100)	52 (96)	12 (100)	4 (100)	0	11 (52)	4 (44)	0	86 (80)	6 (24)
30 kDa	2 (67)	51 (94)	12 (100)	3 (75)	0	5 (24)	1 (11)	0	74 (69)	5 (20)
0-1 band	0	1 (2)	0	0	3 (100)	7 (33)	5 (56)	1 (100)	17 (16)	15 (60)
2 bands	1 (33)	2 (4)	0	1 (25)	0	6 (29)	1 (11)	0	11 (10)	5 (20)
3 bands	0	4 (7)	0	0	0	7 (33)	2 (22)	0	13 (12)	4 (16)
4 bands	2 (67)	47 (87)	12 (100)	3 (75)	0	1 (5)	1 (11)	0	66 (62)	1 (4)
Mean global intensity (±SD)	13.5 ± 3.05				4.4 ± 4.3				10.6 ± 5.5	3.4 ± 4.2
Median global intensity (IQR)	15 (3–16)				5 (0–13)				13 (0–16)	2 (0–16)

^*a*^ CCPA, chronic cavitary pulmonary aspergillosis; CFPA, chronic fibrosing pulmonary aspergillosis; CPA, chronic pulmonary aspergillosis; GM, galactomannan; ICT, immunochromatographic test; IQR, interquartile range; SA, simple aspergilloma; SAIA, subacute invasive aspergillosis; SD, standard deviation; WB, western blot.

^*b*^ CPA patients with consistent and repeated *Aspergillus*-specific IgG levels of <10 AU/mL by ELISA were classified as “seronegative.” These cases were diagnosed as CPA based on positive *Aspergillus *growth in sputum/BALF culture.

### Agreement between *Aspergillus* ICT, WB, and ELISA

Substantial agreement was observed between LDBio ICT and WB across all sera (κ = 0.82; 95% CI: 0.74–0.90), with 91% concordance (Table 5). Among CPA patients, agreement was 88% (κ = 0.64; 95% CI: 0.47–0.81). Heterogeneity was evident across serologic subgroups: seropositive CPA demonstrated 92% concordance but no true agreement due to ceiling effects (κ = –0.02; 95% CI: –0.06 to 0.015), whereas seronegative CPA showed moderate agreement (79%; κ = 0.59; 95% CI: 0.35–0.84). WB exhibited significantly higher sensitivity than ICT in all CPA cases (McNemar’s *P* = 0.003), particularly in seronegative CPA (McNemar’s *P* = 0.016). Sensitivities did not differ significantly between assays in seropositive CPA (McNemar’s *P* = 0.22). Agreement between ICT and ELISA (89.6%, κ = 0.78, 95% CI: 0.69–0.87) and between WB and ELISA (85.6%, κ = 0.71, 95% CI: 0.62–0.81) was similarly high. ICT and ELISA showed 85% agreement in CPA (κ = 0.64, 95% CI: 0.48–0.80), with strong concordance in seropositive but low concordance in seronegative CPA (93.2% vs. 67.6%). Similarly, WB and ELISA demonstrated 82.2% agreement in CPA (κ = 0.53, 95% CI: 0.35–0.70), with near-complete concordance in seropositive (98.6%) but poor agreement in seronegative CPA (47.1%) (Table 5). WB exhibited significantly higher sensitivity than ELISA in detecting *Aspergillus*-specific antibodies (84.1%; 95% CI: 75.8–90.5 vs. 68.2%; 95% CI: 58.5–76.9; McNemar’s *P* = 0.0001).

**TABLE 5:** Agreement between LDBio *Aspergillus* ICT, WB, and ELISA^*a*^^,^^*b*^

Test	Seropositive CPA (*n* = 73)	Seronegative CPA^*c*^ (*n* = 34)	All CPA (*n* = 107)	All sera (*n* = 202)
ICT vs. WB				
% agreement	92	79	88	91
Cohen’s kappa (95% CI)	−0.02 (−0.06, 0.015)	0.59 (0.35, 0.84)	0.64 (0.47, 0.81)	0.82 (0.74, 0.90)
McNemar’s *P*	0.22	**0.016**	**0.003**	**0.0001**
ICT vs. ELISA				
% agreement	93.2	67.6	85	89.6
Cohen’s kappa (95% CI)			0.64 (0.48, 0.80)	0.78 (0.69, 0.87)
McNemar’s *P*			0.21	**0.03**
WB vs. ELISA				
% agreement	98.6	47.1	82.2	85.6
Cohen’s kappa (95% CI)			0.53 (0.35, 0.70)	0.71 (0.92, 0.81)
McNemar’s *P*			**0.0001**	**<0.0001**

^*a*^ CPA, chronic pulmonary aspergillosis; ELISA, enzyme-linked immunosorbent assay; ICT, immunochromatographic test; WB, western blot.

^*b*^ The *P* values in bold indicate statistical significance.

^*c*^ CPA patients with consistent and repeated *Aspergillus*-specific IgG level of <10 AU/mL by ELISA were classified as “seronegative.” These cases were diagnosed as CPA based on positive *Aspergillus *growth in sputum/BALF culture.

### Latent class analysis results

To account for the absence of a perfect reference standard, LCA was performed, assuming conditional independence between ICT and WB (Table 6). The model estimated CPA prevalence at 52% (95% CI: 44–60) within the study population. The ICT demonstrated a sensitivity of 92% (95% CI: 84%–97%) and a specificity of 94% (95% CI: 88%–98%). The WB showed a sensitivity of 87% (95% CI: 77%–94%) and a specificity of 91% (95% CI: 83%–96%).

**TABLE 6:** Diagnostic performance of LDBio *Aspergillus* ICT and WB in CPA using latent class analysis^*a*^

Parameter	% Estimate	95% CI (2.5%–97.5%)
CPA prevalence	52	44, 60
ICT sensitivity	92	84, 97
ICT specificity	94	88, 98
WB sensitivity	87	77, 94
WB specificity	91	83, 96

^*a*^ CPA, chronic pulmonary aspergillosis; ICT, immunochromatographic test; WB, western blot.

### Treatment and outcome

Among CPA patients, 83/107 (78%) received itraconazole-based antifungal therapy. At the time of follow-up, mortality was 1/107 (0.9%), reflecting favorable outcomes with timely and appropriate antifungal treatment.

## DISCUSSION

This study is the first from India to evaluate the diagnostic performance of the LDBio IgG WB assay in CPA. Our findings demonstrate that both the LDBio *Aspergillus* ICT IgG/IgM LFA and LDBio WB IgG assays are highly efficient in diagnosing CPA in post-TB patients, including those with seronegative disease.

The global prevalence of CPA is estimated at 42 per 100,000 population (26), with earlier Indian estimates of 0.02% in 2011 (27) and a revised 5-year TB-associated burden of approximately 1.5 million cases (8). In contrast, reported prevalence is <1 per 100,000 in the United States and Europe, 42.9 per 100,000 in the Democratic Republic of Congo and Nigeria (27), and 5.02 per 100,000 in France (28). As our study was conducted in a selected cohort of symptomatic post-TB patients, prevalence estimates cannot be extrapolated; however, 107/132 (81%) met diagnostic criteria for CPA, and *Aspergillus* IgG seropositivity was detected in 73/132 (55.3%) patients with chronic respiratory symptoms. Comparable high rates have been reported in post-TB cohorts from Vietnam (54.3%) (29), although these exceed the pooled Asian CPA prevalence of 14.7% (30) and regional Indian estimates from Assam (49%) (31) and North India (23%) (32). A recent Indian review estimated the risk of CPA following pulmonary TB to be 10% within the first year, with an additional 1.5% risk over the subsequent 2–5 years (8). High CPA prevalence has also been documented in African TB populations, including Sierra Leone (21%) (33), Kenya (20%) (34), and Ghana (50%) (35).

The demographic and clinical profile of CPA in our cohort closely mirrors previous reports from India (31) and other high-burden TB settings (1, 2), where middle-aged males with a history of smoking or alcohol use predominate. Persistent cough, dyspnea, and hemoptysis were almost universal, highlighting the need for clinicians to maintain a high index of suspicion for CPA in symptomatic, post-TB patients even when microbiological evidence of active TB is lacking, given the close clinical overlap between the two conditions (10, 29, 36). Studies from Vietnam (29), India (37), and Ghana (35) similarly report that more than half of patients presenting with recurrent PTB-like symptoms after documented cure actually have CPA.

We observed a long median interval of 5 years between completion of TB therapy and CPA presentation, which echoes the reports from other high-TB burden countries, including Vietnam, where 73% of patients developed CPA > 5 years after TB treatment (29), and Brazil, where the median interval between PTB and CPA diagnosis was 9 years (38). These data indicate that the interval to CPA development following PTB cure is substantially longer than the interval typically seen in PTB relapse, which may contribute to CPA remaining unrecognized for years. Chronic pulmonary histoplasmosis represents another important differential diagnosis (39). Establishing a definitive diagnosis of CPA is essential, as it can prevent unnecessary exposure to anti-TB therapy and is likely to improve patient outcomes.

High-resolution CT remains the imaging modality of choice for CPA, offering superior delineation of cavities, cavity-wall invasion, intracavitary soft tissue, pleural thickening, and lymphadenopathy (40). In our cohort, the radiologic features were highly discriminatory: fungal balls, cavitary lesions with pericavitary fibrosis, nodules, and bronchiectasis were frequently observed, consistent with established radiologic hallmarks of CPA (10). The high frequency of cavitary lesions, nodules, and mixed radiologic patterns underscores the heterogeneity of CPA and highlights the importance of serologic confirmation, particularly given the nonspecific nature of imaging findings in post-TB lung disease (6, 41).

In our study, LDBio ICT demonstrated high specificity (94.7%) and good sensitivity (73.8%), consistent with existing evaluations of this rapid point-of-care assay (42–45). To date, only a limited number of studies have assessed the diagnostic performance of the LDBio *Aspergillus* WB IgG assay for CPA, reporting sensitivities of 80%–88.6% and specificities of 73.5%–94% (12, 15, 42, 46). In our cohort, LDBio WB exhibited the highest overall sensitivity (84%), and when used in combination with ICT, sensitivity increased to 96%, indicating that a dual-testing strategy may substantially enhance diagnostic accuracy. This contrasts with the findings of Rozaliyani et al. (46), who observed an improvement in specificity (to 81%) without an accompanying increase in sensitivity (80%) when both assays were combined, a discrepancy likely attributable to the smaller number of CPA patients in their study. Youden’s index in our analysis clearly favored the combined use of ICT and WB, supporting its incorporation into diagnostic workflows, particularly in settings where access to confirmatory fluorescent EIA or immunodiffusion testing is limited. In contrast, serum and BALF GM showed poor sensitivity (30% and 36%, respectively) and did not distinguish CPA from non-CPA post-TB lung disease, except in cases of SAIA. We adopted higher GM ODI cutoffs (1.65 for serum and 2.5 for BALF), as proposed by Sehgal et al. (19), to prioritize diagnostic specificity in a post-TB population where structural lung disease and *Aspergillus* airway colonization are common. As anticipated, these thresholds yielded high specificity at the expense of sensitivity, reflecting low or intermittent antigen release in chronic, non-angioinvasive disease. GM positivity was largely confined to subacute invasive presentations, supporting its restricted role in selected clinical phenotypes rather than routine use in CPA diagnosis.

Conventional sputum and BALF cultures exhibit low to moderate sensitivity for the diagnosis of CPA (1), with reported *Aspergillus* culture yields from respiratory specimens ranging from 3.6% to 63% (29, 35, 42, 43, 47). In contrast, our study demonstrated a substantially higher *Aspergillus* isolation rate (80%), exceeding those reported previously. This enhanced recovery likely reflects optimized mycological procedures in our setting, particularly the use of high-volume sputum cultures. Vergidis et al. (22) similarly reported a significantly higher recovery rate of *Aspergillus* spp. with HVCs compared with conventional cultures (54.2% vs. 15.7%). In our cohort, *Aspergillus* spp., particularly *A. niger* and *A. fumigatus*, predominated; however, mixed-species growth was frequent, and non-*Aspergillus* molds were co-isolated in more than one-quarter of CPA cases. Hunter et al. (42) also reported co-isolation of *A. fumigatus* with non-*fumigatus Aspergillus* species in 31.5% of culture-positive CPA cases. These observations have important diagnostic implications, particularly in resource-limited settings where CPA due to non-*fumigatus Aspergillus* species such as *A. niger* and *A. flavus* is relatively common (48, 49). Notably, we observed significantly higher *Aspergillus* IgG concentrations in *A. fumigatus*–associated CPA compared with non-*fumigatus* infections, indicating that negative or low IgG levels do not exclude CPA. This finding underscores the importance of culture-based methods, especially high-volume sputum cultures, in seronegative cases. *Aspergillus* colonization of the lower respiratory tract in patients with obstructive airway disease has been reported in 1%–42% of cases (50), and pulmonary intracavitary colonization with *A. niger* is well recognized among diabetic post-TB patients, where it may lead to CPA complicated by systemic oxalosis (51, 52). Given that many *A. fumigatus* strains implicated in CPA are slow-growing and may exhibit phenotypic variation, sputum cultures may preferentially yield *A. niger* because of its superior colonizing capacity, even when it is not the primary etiologic agent. In our study, both ICT and WB exhibited cross-reactivity with non-*fumigatus Aspergillus* species (*A. niger*, *A. flavus*, *Aspergillus terreus*, and *Aspergillus versicolor*), and with non-*Aspergillus* molds, including *Penicillium* spp. WB also showed cross-reactivity with *F. oxysporum*. Together, these findings highlight critical diagnostic challenges in CPA, including species heterogeneity, mixed fungal colonization, and variable antigenic expression, which may contribute to serologic variability and partially explain the reduced sensitivity of serological assays in non-*fumigatus* infections.

A major strength of this study is the detailed characterization of CPA subtypes and associated serologic profiles. Consistent with the findings of Hunter et al. (42), we observed that seropositive CPA was associated with high ICT reactivity (93%) and universal recognition of key WB bands. In contrast, seronegative CPA, a subgroup traditionally difficult to diagnose, demonstrated substantially higher detection by WB than by ICT (53% vs. 32%). This observation is clinically relevant, as it indicates that WB can capture a broader range of antigenic signatures, including in patients with low IgG titers or infections with non-*fumigatus Aspergillus* species, thereby reducing false-negative results. A study from France similarly reported superior sensitivity and fewer false-negative results for WB compared with the *Aspergillus* immunoprecipitin test (12). The level of agreement between ICT and WB in our cohort (κ = 0.82) was similar to that reported by Rozaliyani et al. (κ = 0.72) (46), indicating that either test may be appropriate for routine screening, although WB consistently outperformed ICT in seronegative CPA and mixed-species infections. The strong concordance observed with *Aspergillus*-specific IgG ELISA further supports WB as a robust and reliable serological tool for the diagnosis of CPA.

For the first time, we evaluated the diagnostic performance of LDBio *Aspergillus* WB IgG assay across distinct CPA subtypes. The ICT performed well in CCPA and CFPA, consistent with the high sensitivities previously reported by Sehgal et al. (85% in CCPA and 93.3% in CFPA) (44). Notably, the WB assay maintained high sensitivity across all CPA subtypes, underscoring its versatility across diverse clinical presentations. The WB banding patterns provided further immunodiagnostic insight. Dominant recognition of the 16 kDa, 18–20 kDa, and 22 kDa antigens in CPA aligns with previous proteomic analyses of *Aspergillus* immunodominant proteins (12). Moreover, the pronounced multi-band reactivity observed in seropositive CPA, in contrast to weak or absent reactivity in seronegative CPA and non-CPA patients, suggests that WB not only aids in CPA diagnosis but also provides semi-quantitative information on host immune recognition of *Aspergillus* antigens, potentially serving as an adjunctive marker of disease activity or fungal burden. A similar semi-quantitative interpretation of WB results based on band number and intensity was also employed by Oliva et al. for CPA diagnosis (12).

Given the absence of a universally accepted gold standard serological test for CPA (12), our LCA provides an unbiased estimate of performance. The analysis yielded high sensitivity (92%) and specificity (94%) for ICT, with similarly robust estimates for WB (sensitivity 87% and specificity 91%), indicating that both tests retain diagnostic accuracy independent of culture- or ELISA-based classification. These results suggest that the LDBio ICT and WB assays capture complementary serologic dimensions, remaining reliable even when conventional criteria are insufficient to distinguish CPA from other post-TB lung disease phenotypes.

Several observations from this study hold significant practical value. The high CPA seropositivity among symptomatic post-TB patients indicates an urgent need for routine CPA evaluation within post-TB lung disease care pathways, particularly in countries like India with large populations of TB survivors. The high specificity, low cost, rapid turnaround, and operational feasibility of ICT make it suitable as a first-line screening tool in peripheral and district-level facilities. The enhanced sensitivity and detailed antigenic profiles provided by WB justify its use as a second-line confirmatory test in referral centers, particularly in seronegative and culture-negative cases or when species other than *A. fumigatus* are implicated. Our findings also reinforce that serum and BALF GM should not be used as a primary diagnostic tool for CPA, except in selected subacute invasive presentations in immunocompromised patients. The favorable clinical outcomes and low mortality observed in our cohort following itraconazole therapy highlight the substantial potential for morbidity reduction with timely CPA diagnosis.

The key strengths of this study include its prospective design, comprehensive clinico-radiologic characterization, incorporation of multiple microbiological modalities alongside serology for CPA diagnosis, and application of LCA to evaluate the diagnostic performance of the LDBio ICT and WB assays. However, several limitations should be acknowledged. The single-center design and potential referral bias may limit generalizability, and the study did not include longitudinal assessment of serologic responses to therapy. Additionally, only smear-negative, GeneXpert-negative post-TB patients with persistent respiratory symptoms were evaluated, which restricts applicability to individuals with other underlying lung diseases who are also at risk for CPA. The high CPA seropositivity in our cohort may have modestly inflated positive predictive metrics, underscoring the need for multicentric validation across more diverse post-TB lung disease populations.

### Conclusion

The LDBio ICT and WB assays demonstrate excellent diagnostic performance for CPA in post-TB patients, with their combined use offering superior sensitivity, accuracy, and discriminatory power. The WB assay improves detection in seronegative and non-*fumigatus Aspergillus* infections, highlighting its usefulness in complex cases. Distinct banding profiles and intensity patterns further support the role of WB as a semi-quantitative tool reflecting host immune response to *Aspergillus* antigens. Integration of these assays into post-TB lung disease care pathways has the potential to substantially improve CPA diagnosis, reduce delays in antifungal treatment, and mitigate long-term morbidity in TB-endemic regions. Our findings inform diagnostic strategies and could facilitate the development of standardized serological criteria for CPA in high-risk populations.

## Data Availability

The data that support the findings of this study are available from the corresponding author upon reasonable request.
